# Within-Leaf Nitrogen Allocation in Adaptation to Low Nitrogen Supply in Maize during Grain-Filling Stage

**DOI:** 10.3389/fpls.2016.00699

**Published:** 2016-05-24

**Authors:** Xiaohuan Mu, Qinwu Chen, Fanjun Chen, Lixing Yuan, Guohua Mi

**Affiliations:** Center for Resources, Environment and Food Security, College of Resources and Environmental Science, China Agricultural UniversityBeijing, China

**Keywords:** bioenergetics, light harvesting, phosphoenolpyruvate carboxylase, photosynthetic rate, photosynthetic nitrogen use efficiency, pyruvate orthophosphate dikinase, ribulose-1, 5-bisphosphate carboxylase, thylakoid nitrogen

## Abstract

Nitrogen (N) plays a vital role in photosynthesis and crop productivity. Maize plants may be able to increase physiological N utilization efficiency (NUtE) under low-N stress by increasing photosynthetic rate (*P*_n_) per unit leaf N, that is, photosynthetic N-use efficiency (PNUE). In this study, we analyzed the relationship between PNUE and N allocation in maize ear-leaves during the grain-filling stage under low N (no N application) and high N (180 kg N ha^-1^) in a 2-year field experiment. Under low N, grain yield decreased while NUtE increased. Low-N treatment reduced the specific N content of ear leaves by 38% without significant influencing *P*_n_, thereby increasing PNUE by 54%. Under low-N stress, maize plants tended to invest relatively more N into bioenergetics to sustain electron transport. In contrast, N allocated to chlorophyll and light-harvesting proteins was reduced to control excess electron production. Soluble proteins were reduced to shrink the N storage reservoir. We conclude that optimization of N allocation within leaves is a key adaptive mechanism to maximize *P*_n_ and crop productivity when N is limited during the grain-filling stage in maize under low-N conditions.

## Introduction

In modern crop production systems, nitrogen (N) plays a vital role in yield formation; it is the mineral element required in the greatest amounts by plants, and is often the growth-limiting nutrient. N is a fundamental constituent of many cell components. In leaves, forms of N include soluble components such as nitrates, amino acids and proteins, and insoluble components in cell walls, membranes and other structures. N used in the photosynthetic apparatus can be divided into two categories, namely, that associated with photosynthetic enzymes and thylakoid N. The main photosynthetic enzymes, ribulose-1,5-bisphosphate carboxylase (Rubisco), phosphoenolpyruvate carboxylase (PEPC) and pyruvate orthophosphate dikinase (PPDK), are involved in carbon reduction reactions and are the most abundant enzymes in photosynthesis (Tazoe et al., 2005). Thylakoid N is distributed between two types of proteins: (1) proteins related to bioenergetics, including Cyt *b/f* and CF_1_/CF_0_ involved in electron transport and photophosphorylation, and (2) light-harvesting proteins, such as photosystem I (PSI), photosystem II (PSII), and light-harvesting complex II (LHCII) proteins, that are associated with the light reactions of photosynthesis (Makino et al., 1997; Takashima et al., 2004; Tazoe et al., 2005; Uribelarrea et al., 2009). When grown under high light, C_3_ leaves typically invested 58% of leaf N to soluble protein (about 40% of which is Rubisco) and 22% to thylakoids (Evans, 1989a; Poorter and Evans, 1998; Makino et al., 2003). While in C_4_ plant, about 45% leaf N partitioned into soluble protein (about 20% of which is Rubisco) and 28% into thylakoids (Makino et al., 2003; Ghannoum et al., 2005; Tazoe et al., 2005). And about 80% of thylakoids N in C_3_ plants and 75% of thylakoids N in C_4_ plants allocated in light-harvesting proteins, the other allocated in bioenergetics (Evans, 1989b; Makino et al., 2003). A strongly positive correlation has been widely reported between photosynthetic capacity and N content per unit leaf area (Makino et al., 2003; Takashima et al., 2004; Tazoe et al., 2005). N supply thus has substantial effects on plant growth and development and yield formation.

Under N-limited conditions, maize plants increased N use efficiency by increasing N uptake efficiency (NUpE) and/or N utilization efficiency (NUtE; Moll et al., 1982; Hirel et al., 2007). Efficient N acquisition is believed to rely on a large and deep root system (Lawlor, 2002; Wang et al., 2005; Mi et al., 2010; Lynch, 2013). NUtE, which is defined as grain yield produced per unit of plant N (Moll et al., 1982; Hirel et al., 2007), is an indicator of how plant N is used for photosynthetic production. On a whole-plant level, an optimal N distribution in the plant canopy may improve photosynthesis without additional N input (Dwyer and Stewart, 1986; Drouet and Bonhomme, 1999; Hikosaka, 2014). How N can be efficiently used to produce as much photosynthate in single leaves, especially under N-limited conditions, is less clear. Photosynthetic N use efficiency (PNUE), the rate of photosynthesis per unit leaf N, is increased under low-N stress (Sage and Pearcy, 1987; Ghannoum et al., 2005; Uribelarrea et al., 2009; Hawkesford, 2012). In a recent field study, an N-efficient maize genotype was found to have a higher PNUE than an inefficient one (Chen et al., 2014). In spite of these findings, the underlying physiological mechanism of photosynthetic N use efficiency remains unclear.

In general, C_4_ plants exhibit a greater PNUE than C_3_ plants (Brown, 1978; Schmitt and Edwards, 1981; Sage and Pearcy, 1987, 2000; Makino et al., 2003). This higher PNUE is mainly because C_4_ plants can eliminate photorespiration by increasing CO_2_ levels in the vicinity of Rubisco. In maize, a C_4_ plant, the N cost for C_4_-cycle enzymes (PPDK and PEPC) is not large; in addition, the lower amount of Rubisco in maize allows a greater N investment to be made in the thylakoid components compared with rice, a C_3_ plant (Makino et al., 2003). And NADP-malic enzyme (ME) C_4_ grasses have a higher PNUE and NUE than NAD-ME C_4_ grasses (Ghannoum et al., 2005). The main reason is that they have less leaf N and soluble protein but a faster *k*_cat_ of Rubisco in NADP-malic enzyme (ME) C_4_ grasses (Ghannoum et al., 2005).

In maize, around 80–100% of dry matter for grain yield formation is contributed by post-silking photosynthesis (Tollenaar and Lee, 2006; Chen et al., 2013, 2014; Kosgey et al., 2013; Antonietta et al., 2014). During the post-silking period, the maintenance of a high rate of whole-plant photosynthesis is difficult, as N is continuously remobilized from the leaves, especially under N-limited conditions. An increase in PNUE would potentially resolve this problem (Chen et al., 2014). Most of the research on PNUE has been conducted at seedling stage (Sugiharto et al., 1990; McCullough et al., 1994; Makino et al., 2003), less is known about the physiological determinants of PNUE during the grain-filling stage under field conditions. The aim of this study was consequently to uncover the relationship between within-leaf N allocation and PNUE.

## Materials and Methods

### Plant Materials and Growth Conditions

Field experiments were conducted in 2013 and 2014 at the Shangzhuang Experimental Station, China Agricultural University, Beijing, China (116°11′ N, 40°8′ E). The field soil was a typical Ustochrept soil with the following physical and chemical characteristics (0–20 cm) at the start of the experiment: 92.4 kg N ha^-1^ CaCl_2_-extracted mineral N (N_min_), 16.2 mg kg^-1^ available phosphorus (Olsen-P), 122.6 mg kg^-1^ammonium acetate extractable potassium (K), 11.6 g kg^-1^ organic matter, and a pH (H_2_O) of 8.0. Before plowing, the field was irrigated, plowed (immediately before plowing) and sprinkled with a base fertilizer consisting of 135 kg P_2_O_5_ ha^-1^ [as superphosphate (Ca (H_2_PO_4_)_2_⋅H_2_O)] and 75 kg K_2_O ha^-1^ (as K_2_SO_4_). Additional N was applied at two different treatment levels: 180 kg N ha^-1^ (HN) and no N application (LN). For the HN treatments, 30% of the N fertilizer was applied before sowing and the remaining at the V6 stage (six expanded leaves). In the previous research conducted in the same field, N application at 180 kg ha^-1^ was found sufficient to achieve the maximum yield (data not shown). At harvest, the residual soil N_min_ (0–20 cm) was 52 and 38 kg N ha^-1^ in 2013 and 2014, respectively.

The experiment consisted of a randomized block design with four replicates, with each plot 15 m long and 9 m wide. Zhengdan 958, the most popular commercial hybrid across North and Northeast China, was sown on April 28, 2013, and April 29, 2014, and was harvested on September 7 and September 3 of 2013 and 2014, respectively. The plots were over-seeded using a hand planter, and then thinned at the seeding stage to 60,000 plants ha^-1^. Distances between rows and plants were 60 and 28 cm, respectively. Plots were kept free of weeds, insects and diseases during the growth season. During 2013, rainfall was adequate during the entire growth period and no irrigation was applied. Because a severe drought occurred during the silking stage in 2014, irrigation was applied during that period to ensure normal plant growth.

### Gas Exchange and Chlorophyll Fluorescence Parameters Measurements

At the silking stage, six plants with the same silking date were tagged. At 20 and 23 days after silking in 2013 and 2014, respectively, the ear-leaf net photosynthetic rate (*P*_n_) of six tagged plants per plot was determined. The average of the *P*_n_ values of the six plants in each plot was taken as a replicate. *P*_n_ was measured with a portable photosynthesis system (Li6400; LI-COR, Lincoln, NE, USA) coupled to a standard red/blue LED broadleaf cuvette (6400-02B; LI-COR) and a CO_2_ mixer (6400-01; LI-COR) at a light intensity of 1,600 μmol m^-2^ s^-1^. Measurements were obtained at a leaf temperature of 30 ± 0.5°C and a CO_2_ concentration inside the chamber of 400 ± 1 μmol CO_2_ (mol air)^-1^ (Ding et al., 2005a; Uribelarrea et al., 2009). The following day, the same plants that had been used for the photosynthetic rate measurements in each plot were used to obtain chlorophyll fluorescence using Li6400. The plants were continuously illuminated at least 1 h. CO_2_ concentration inside the chamber was 400 μmol mol^-1^ and holding incident irradiance at 1600 μmol m^-2^ s^-1^. The actual quantum yield of PSII photochemistry (ΦPSII) and electron transport rate (ETR, μmol e^-^ s^-1^ m^-2^) were calculated as defined by Ding et al. (2005b). Leaf area was calculated according to Sanderson et al. (1981) as leaf length × maximum width × k, where k is a shape factor equal to 0.75 (Birch et al., 2003).

### Biochemical Measurements

After measurement of chlorophyll fluorescence parameters, two leaves per plot were removed, immediately frozen in liquid N_2_ and stored at -80°C for subsequent analysis. Two additional leaves were dried at 65°C to a constant weight. After removal of midribs, the dried leaves were thoroughly ground and then analyzed for total N with an NC analyzer (Vario EL III; Elementar, Hanau, Germany). Specific leaf N (SLN) was calculated as N content per unit leaf area.

Rubisco, PEPC, and PPDK contents of frozen leaves were determined using the method of Makino et al. (2003) with minor modifications. Using a chilled mortar and pestle, the frozen leaves were homogenized in extraction buffer containing 0.2 mM EDTA-Na_2_, 10 mM dithiothreitol, 2 mM iodoacetic acid, 0.1% Triton X-100 and 100 mM Tris-HCl at pH 6.8. The homogenate was centrifuged at 12,000 *g* for 20 min at 4°C. A portion of the supernatant was used for determination of total soluble protein content using a protein assay kit based on the Bradford method (Bio-Rad Protein Assay; Bio-Rad, CA, USA). The supernatant was treated with double-strength loading buffer [5% (w/v) lithium dodecyl sulfate, 5% β-mercaptoethanol, 0.1% bromophenol blue, 5% glycerol and 25 mM Tris-HCl at pH 6.8], heated at 100°C for 5 min, and analyzed by sodium dodecyl sulfate (SDS)-polyacrylamide gel electrophoresis. Rubisco, PEPC, and PPDK were determined spectrophotometrically by formamide extraction of their Coomassie-Brilliant-Blue-R-250-stained bands, which corresponded to 99 kDa for PEPC (Uedan and Sugiyama, 1976), 94 kDa for PPDK (Sugiyama, 1973) and 52 and 15 kDa for Rubisco (Makino et al., 2003). Protein concentration was calculated using bovine serum albumin as a standard (Makino et al., 1986; Fukayama et al., 2001). Cell wall biomass and N content were analyzed according to Onoda et al. (2004) with modifications. The pellet was resuspended with Tris-HCl buffer containing 3% (w/v) SDS, incubated at 90°C for 5 min, and then centrifuged at 2,500 *g* for 5 min. This procedure was repeated eight times. To remove small amounts of cytoplasmic protein contamination, the pellet was washed six times with 0.2 M KOH followed by centrifugation at 2,500 *g* for 5 min. These procedures removed proteins weakly bound to cell walls, leaving behind only tightly bound (structural) proteins. After six washes with distilled water and six washes with ethanol, the tube containing the pellet was dried in an oven at 75°C to a constant weight. After drying, the cell wall materials were weighed and their total N concentrations were determined using the NC analyzer.

Chlorophyll (Chl) was extracted from leaf disks with acetone and ethanol. The absorbance of the extracts was spectrophotometrically measured at 645 and 663 nm (Porra et al., 1989). Nitrate was extracted from fresh leaves with distilled water and quantified following Cataldo et al. (1975). Free amino acids were determined according to Moore (1968).

Thylakoid membranes were prepared according to standard methods (Zhang et al., 1999; Peng et al., 2006). The leaves were homogenized with a chilled mortar and pestle in an ice-cold isolation buffer containing 400 mM sucrose, 50 mM HEPES-KOH (pH 7.8), 10 mM NaCl, and 2 mM MgCl_2_ and filtrated through four layers of cheesecloth. The filtrate was centrifuged at 5000 *g* for 10 min. The thylakoid pellets were washed with isolation buffer, recentrifuged, and suspended in isolation buffer. The thylakoid membrane pellets were dried in an oven at 75°C to a constant weight. After drying, the thylakoid membrane was weighed and their total N concentrations were determined using the NC analyzer.

### Yield, N Uptake and Physiological N Utilization Efficiency (NUtE)

At silking and physiological maturity, five consecutive plants per plot were cut at the soil surface and separated into leaves, stalks (including leaf sheaths and tassels), husks/cobs and grains. All samples were dried at 65°C to a constant weight. The silking stage was considered to be when 50% of the ears in a plot attained silking, whereas physiological maturity corresponded to the stage when a black layer was visible at the grain base in 50% of the ears. Dry samples were weighed and ground to a powder; N concentrations were determined using the semi-micro-Kjeldahl method. NUtE values were calculated as grain dry weight divided by total N content per plant. At physiological maturity, two rows of plants in each plot were harvested for determination of grain yield. Grain yield was standardized to 14% moisture.

### Calculations

Thylakoid N can be divided into two categories according to its allocation: (1) bioenergetics (associated with the electron transport chain and photophosphorylation) and (2) light-harvesting (involved in PSI, PSII, and LHC). N associated with light harvesting (N_h_) was calculated assuming 37 mol mol^-1^Chl (Ghannoum et al., 2005), while N in bioenergetics (N_b_) was calculated as N_TH_ minus N_h_. N allocated to Rubisco, PEPC, PPDK, and soluble protein was calculated assuming 16% N in proteins (Ghannoum et al., 2005).

### Statistical Analysis

Data across N treatments and years were first pooled and subjected to a two-factor analysis of variance using the ANOVA procedure implemented in SPSS Statistics 17.0 (SPSS, Inc., Chicago, IL, USA). Differences were compared using the least significant difference test at a 0.05 level of probability. It is found that the N treatment × Year interaction effect is not significant for almost all the measured parameters (Supplemental Table S1). Therefore, the 2 years’ data was averaged for each parameter. All figures were constructed using GraphPad Prism 5 (GraphPad Software Inc., 2007).

## Results

### Effect of N Supply on Grain Yield, N Accumulation, NUtE, *P*_n_, and PNUE

Under LN stress, grain yield and total N uptake decreased by 32 and 57%, respectively (**Table 2**). In contrast, physiological N utilization efficiency (NUtE) increased by 57% compared with HN treatment.

**Table 1:** Abbreviations, symbols used and their units used in the text.

Symbol	Definition	Units
3-PGA	3-phosphoglyceric acid	–
CA	Carbonic anhydrase	–
Chl	Chlorophyll	μmol m^-2^
Chl *a*	Chlorophyll *a*	μmol m^-2^
Chl *b*	Chlorophyll *b*	μmol m^-2^
Fd	Ferredoxin	-
g_bs_	Bundle-sheath conductance	-
LHCII	Light harvest complex	-
LUS	Rubisco large subunits	-
Mal	Malate	-
MDH	Malate dehydrogenase	-
N	Nitrogen	-
N_a_	Nitrogen in amino acids	mg m^-2^
N_b_	Nitrogen in bioenergetic protein	mg m^-2^
N_b_/N_TH_	Fraction of thylakoid nitrogen allocated to bioenergetics	%
N_cw_	Nitrogen in cell wall	mg m^-2^
N_h_	Nitrogen in light-harvesting protein	mg m^-2^
N_h_/N_TH_	Fraction of thylakoid nitrogen allocated to light-harvesting protein	%
ns	Not significant	-
N_s_	Nitrogen in the soluble proteins other than PEPC, PPDK, and Rubisco	mg m^-2^
N_T_	Nitrogen in thylakoid	mg m^-2^
N_TH_/N	Fraction of nitrogen allocated to thylakoid	%
OAA	Oxaloacetic acid	-
PC	Plastocyanin	-
PEPC	Phosphoenolpyruvate carboxylase	-
*P*_n_	Photosynthetic rate	μmol CO_2_ m^-2^ s^-1^
*P*_max_	Maximum photosynthetic rate	μmol CO_2_ m^-2^ s^-1^
PNUE	Photosynthetic nitrogen use efficiency	μmol CO_2_ g^-1^N s^-1^
PPDK	Pyruvate orthophosphate dikinase	-
PQ	Plastoquinone	-
PSI	Photosystem I	-
PSII	Photosystem II	-
Pyr	Pyruvate	-
Rubisco	Ribulose 1,5-bisphosphate carboxylase/oxygenase	-
RuBP	Ribulose 1,5-bisphosphate	-
SLN	Specific leaf nitrogen	g m^-2^
SSU	Rubisco small subunits	-
Triose-P	Triose-phosphate	-

**Table 2:** Effect of nitrogen (N) supply on grain yield, N accumulation at maturity and N utilization efficiency (NUtE).

N treatments	Grain yield (kg ha^-1^)	N accumulation in maturity (g plant^-1^)	NUtE [g grain (gN)^-1^]
HN	9827a	4.49b	36.8b
LN	6710b	1.94a	57.9a

*Data are the means for the 2 years. Numbers followed by different letters indicate significant differences (*P* < 0.05).*

For the ear-leaf, LN treatment significantly affected SLN, but had little influence on *P*_n_ (**Table 3**). As a calculated result, PNUE was increased in the LN treatment. Compared with the HN treatment, SLN was 38% lower and PNUE was 54% higher under the LN treatment.

**Table 3:** Effect of nitrogen (N) supply on net photosynthetic rate (*P*_n_), specific leaf N (SLN) and photosynthetic N use efficiency (PNUE) in maize ear-leaves during the grain-filling stage.

N treatments	*P*_n_ μmol CO_2_ m^-2^s^-1^	SLN g m^-2^	PNUE μmol CO_2_ g N^-2^s^-1^
HN	31.6a	2.48a	12.98c
LN	30.5a	1.55c	19.69a

*P_n_ was measured using a light intensity of 1600 μmol m^-2^ s^-1^. Measurements were made on ear-leaves between 09:00 and 12:00. Data are the means for the 2 years. Numbers followed by different letters indicate significant differences (*P* < 0.05).*

### Effect of N Supply on N Investment into Soluble-N Components

Nitrate content in LN-treated plants was higher than in the HN treatment (**Table 4**). Leaf nitrate content was very low, accounting for about 1% of total leaf N (**Figure 3**). The amount of N in amino acids was 51% lower under the LN treatment compared with HN treatment (**Table 4**).

**Table 4:** Effect of nitrogen (N) supply on the contents of N compounds in maize ear-leaves during the grain-filling stage.

	N treatment		
Parameter		HN	LN
Nitrate	mg m^-2^	22b	26a
Soluble protein	mg m^-2^	885a	447b
PEPC	mg m^-2^	85a	39b
PPDK	mg m^-2^	68a	33b
Rubisco	mg m^-2^	355a	170b
N_TH_	mg m^-2^	775a	452b
N_b_	mg m^-2^	107a	101a
N_h_	mg m^-2^	668a	351b
N_cw_	mg m^-2^	243a	158b
N_a_	mg m^-2^	112a	55b

*Data are the means for the 2 years. Numbers followed by different letters indicate significant differences (*P* < 0.05). Soluble protein included PEPC, PPDK, and Rubisco. Abbreviations were listed in **Table 1**.*

Soluble protein content was almost twofold less in plants grown under the LN treatment conditions (**Table 4**). Low N reduced the contents of Rubisco (including large and small subunits), PEPC and PPDK per unit leaf area by 52.0, 54.2, and 51.0%, respectively (**Table 4**, **Figure 4**; **Supplementary Figure S1**). When calculated on the basis of leaf N, Rubisco, PEPC, and PPDK concentrations per leaf N were, respectively, 24, 28, and 23% lower under LN treatment than under HN treatment (data not shown).

### Effect of N Supply on the N Allocation into Structure-N Components

**FIGURE 1:**
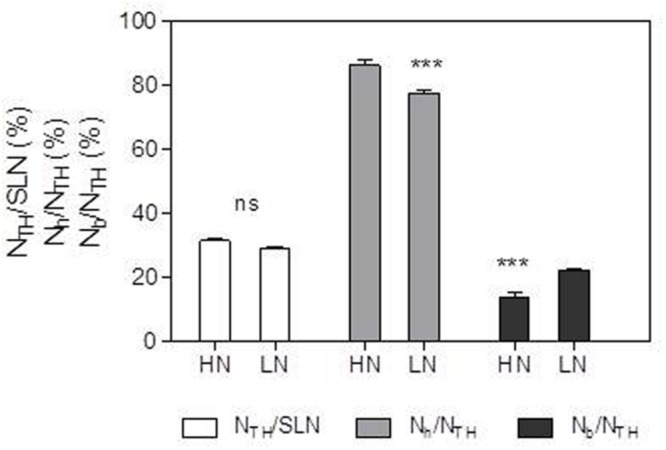
**Effect of nitrogen (N) supply on the percentages of N_TH_/specific leaf N (SLN), N_h_/N_TH_ and N_b_/N_TH_ of ear-leaf in maize.** Bars denote the SE of the mean. ns, not significant (*P* > 0.05); ^∗^*P* < 0.05, ^∗∗^*P* < 0.01, ^∗∗∗^*P* < 0.001, respectively. HN corresponds to a N application of 180 kg ha^-1^; LN indicates that no N was applied.

N_TH_ and N_h_ expressed per unit leaf area were 44.6 and 47.4% lower, respectively, in the LN treatment compared with HN (**Table 4**; **Figure 4**). There was no significant difference in N_b_ between N treatments. Compared with HN treatment, N_h_/N_TH_ was reduced and N_b_/N_TH_ was increased under LN treatment, while N_TH_/SLN was no significant difference (**Figures 1** and **4**).

**FIGURE 2:**
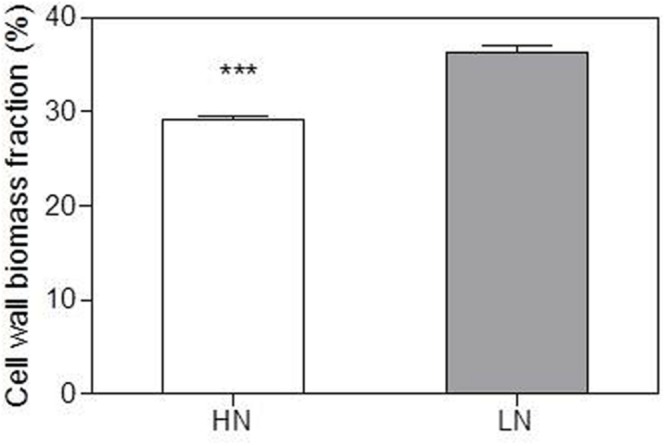
**Effect of nitrogen (N) supply on the cell wall biomass fraction of ear-leaf in maize.** Bars denote the SE of the mean. ns, not significant (*P* > 0.05); ^∗^*P* < 0.05, ^∗∗^*P* < 0.01, ^∗∗∗^*P* < 0.001, respectively. HN corresponds to a N application of 180 kg ha^-1^; LN indicates that no N was applied.

Ear-leaf cell-wall N content (N_cw_) was 35% lowers in LN compared with the HN treatment (**Table 4**). However, the fraction of cell wall biomass was higher in the LN treatment (**Figure 2**).

### Effect of N Supply on the Leaf N Budget

**FIGURE 3:**
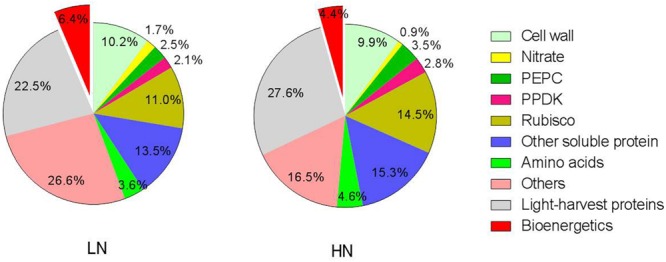
**Effect of nitrogen (N) supply on the percentage of different N components in total ear-leaf N.** Exploded slices were significantly different between high and low N supply at *P* < 0.05.

The percentage of leaf N allocated to different N components is summarized in **Figure 3**. Relative to HN treatment levels, the LN treatment significantly reduced the percentage of N allocated to soluble proteins (including Rubisco, PEPC, and PPDK) and light-harvesting proteins. Among the three major enzymes—Rubisco, PEPC, and PPDK —the percentage of leaf N allocated to Rubisco, PEPC, and PPDK declined 24, 28, and 23%, respectively, under LN treatment. In contrast, N allocation to bioenergetics processes increased by 62.5% under low N (4.0% at HN and 6.5% at LN). Unexpectedly, the allocation of N to nitrate and the remaining N components were also higher under low N. The proportion of N in amino acid was lower 22% under LN treatment. The percentage of N allocated to cell walls was similar between N treatments.

**FIGURE 4:**
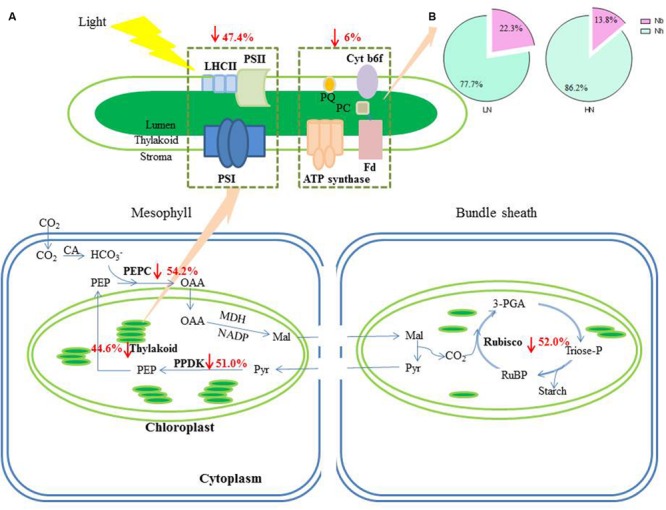
**The change of N contents in photosynthetic apparatus caused by LN treatment. (A)** The percentage together with the arrows indicate the reduction of N in different photosynthetic apparatus under LN compared to HN treatment. **(B)** The allocation of N between N_h_ and N_b_ within the thylakoid under LN and HN treatment. Abbreviations were listed in **Table 1**.

### Effect of N Supply on Chlorophyll (Chl) and Chlorophyll Fluorescence

**FIGURE 5:**
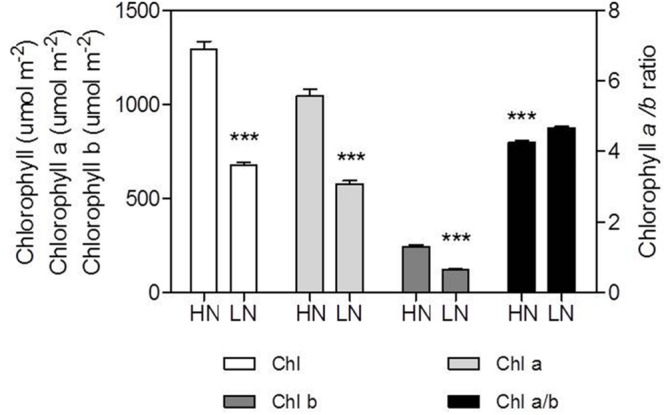
**Effect of nitrogen (N) supply on the content of chlorophyll (Chl), Chl *a*, Chl *b*, and Chl *a/b* ratio of ear-leaf in maize.** Bars denote the SE of the mean. ns, not significant (*P* > 0.05); ^∗^*P* < 0.05, ^∗∗^*P* < 0.01, ^∗∗∗^*P* < 0.001, respectively. HN corresponds to a N application of 180 kg ha^-1^; LN indicates that no N was applied.

**FIGURE 6:**
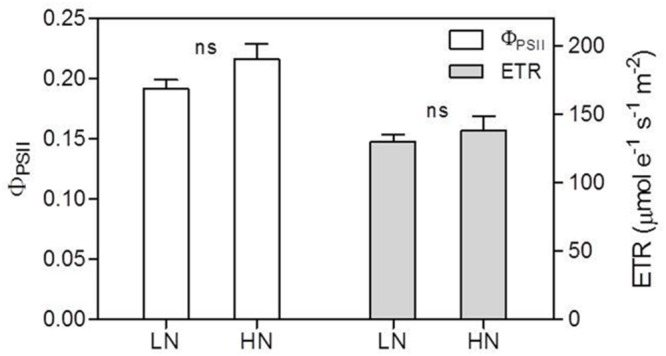
**Effect of nitrogen (N) supply on the actual quantum yield of PSII photochemistry (ΦPSII) and electron transport rate (ETR) of ear-leaves in maize.** Bars denote the SE of the mean. ns, not significant (*P* > 0.05); ^∗^*P* < 0.05, ^∗∗^*P* < 0.01, ^∗∗∗^*P* < 0.001, respectively. HN corresponds to a N application of 180 kg ha^-1^; LN indicates that no N was applied.

The contents of Chl, Chl *a* and Chl *b* per unit leaf area were reduced by LN treatment (**Figure 5**). Chl content decreased by 47% in LN leaves than in HN leaves; conversely, the Chl *a/b* ratio was significantly higher in LN leaves than in HN leaves (**Figure 5**). There were no significant differences in ΦPSII and ETR between N treatment (**Figure 6**).

## Discussion

As is well documented, low-N stress causes stunted plant growth by reducing leaf expansion and *P*_n_ (Radin and Boyer, 1982; Snir and Neumann, 1997; Gastal and Lemaire, 2002; Jovanovic et al., 2004; KavanovA et al., 2008). Reduction in leaf expansion (leaf area) helps sustain leaf N concentration, thereby contributing to the maintenance of *P*_n_ (Radin and Boyer, 1982; Gastal and Lemaire, 2002) this seems to be especially true in N-efficient maize genotypes (McCullough et al., 1994). Under the experimental conditions of our study, N deficiency caused a relative reduction of 36% in ear-leaf leaf area index (data not shown) and 38% in SLN, from 2.48 g m^-2^ under HN treatment to 1.55 g m^-2^ under LN treatment. *P*_n_, however, was not significantly affected. Thus, PNUE was greatly increased, by 54%, under the LN treatment. According to Sinclair and Horie (1989), for each N allocation to leaves, an optimum N content exists to maximize crop biomass production. The optimal SLN for maximum *P*_n_ has been reported to be approximately 1.5 in field-grown maize (McCullough et al., 1994; Muchow and Sinclair, 1994; Paponov et al., 2005; Vos et al., 2005), which fits well with the value obtained under LN treatment in the present study. A higher PNUE enables maize plants to use N efficiently for biomass production and to increase N use efficiency (**Table 2**). A genotypic difference in PNUE has in fact been observed in maize, with N-efficient genotypes having higher PNUE than inefficient ones (McCullough et al., 1994; Paponov and Engels, 2003; Echarte et al., 2008; Chen et al., 2014). This variation suggests that genetic improvement of PNUE is possible, and elucidation of the physiological mechanism underlying high PNUE is therefore essential. Because the high PNUE observed under LN treatment contributed to a decrease in SLN rather than an increase in *P*_n_, within-leaf optimization of N allocation potentially explains high PNUE.

In accord with previous studies (Pons and Westbeek, 2004; Ding et al., 2005a), LN treatment led to reduced total Chl, Chl *a* and Chl *b* contents (**Figure 5**). A reduction in Chl, together with an increase in zeaxanthin formation and the capacity for excitation energy dissipation in PSII (Khamis et al., 1990; Lu et al., 2001), are believed to be strategies for protection of PSII function (Khamis et al., 1990). The lower capacity of *P*_n_ under low-N conditions means that the greater excess of excitation energy may potentially lead to increased susceptibility of PSII to photo-inhibition (Ramalho et al., 1997; Grassi et al., 2001; Lu et al., 2001). Although the degree of low-N stress in our study was insufficient to reduce leaf *P*_n_, the leaves responded by reducing Chl content to avoid excess production of excitation energy. The change in N distribution within thylakoids was consistent with this change in Chl. Although N_TH_ in the LN treatment was less than that in the HN treatment (**Table 4**), the percentage of N allocated to thylakoids was similar between N rates (30–31%; **Figure 1**). These values are close to those uncovered by Makino et al. (2003), who reported that 34% of leaf N was allocated to thylakoids in their studied maize plants. There are two types of thylakoid N, namely that associated with bioenergetics such as the electron transport chain and photophosphorylation, and N involved in the light-harvesting complex. Further analysis indicated that the distribution of N between these two thylakoid categories differed between N treatments. In particular, relatively more N of thylakoid was allocated into biogenetics than into light harvesting under LN compared with the HN treatment (**Figures 1** and **4**). In fact, absolute N content devoted to bioenergetics was similar under the LN and HN treatment (**Table 4**). This proved that leaf prioritization for stabilization of electron transfer photophosphorylation under low-N stress and thus maximization of quantum yield (Khamis et al., 1990; Lu and Zhang, 2000; Lu et al., 2001; Tóth et al., 2002; Antal et al., 2010). This is supported by the finding that Φ_PSII_ and ETR were not different between N treatments (**Figure 6**). On the other hand, a reduction in the allocation of N to the light-harvesting complex should help control excess electron production. In C_3_ plants, Hikosaka and Terashima (1995) used models to calculate the nitrogen allocation among photosynthetic components at different incident photon flux density and N levels. They found that the relative amount of N in Rubisco decreases with decreases in leaf N, while that in electron transport components, coupling factor and Calvin cycle enzymes increases (apart from Rubisco, PSI, and PSII). And the relative amount of N partitioned into Chl-protein complexes (PSI, PSII, and LHCII) remained almost constant with decreasing leaf N.

Low N supply caused a relative reduction in both soluble protein per unit leaf-N content (from 2.23 to 1.80 g g^-1^ N) and the percentage of total soluble protein-N in total leaf N (from 36% to 30%; **Figure 3**). This latter value is in good agreement with the results of Makino et al. (2003), who reported that 33% of leaf N was allocated to soluble protein. Among soluble proteins, Rubisco, PEPC, and PPDK are key enzymes involved in C_4_ photosynthesis. Sugiyama et al. (1984) found that the proportions of Rubisco, PEPC, and PPDK in soluble protein were, respectively, about 35, 8, and 6% in leaves of maize seedlings grown under optimal conditions. At near-optimal growth conditions in our study, Rubisco, PEPC, and PPDK constituted approximately 40, 10, and 8% of soluble protein and 14.5, 3.5, and 2.8% of total leaf N, respectively (**Table 4**; **Figure 3**). Under LN treatment, the contents of Rubisco, PEPC, and PPDK per unit leaf area were reduced by 52, 54, and 51%, respectively (**Table 4**; **Figure 4**). In addition, their corresponding proportions of total leaf N declined by 24, 28, and 23% under low N condition (**Figure 3**). These responses are not consistent with what found in the developed young leaves at seedling stage. In these leaves, low N availability preferentially reduced PEPC, followed by PPDK, with less effect on Rubisco. As a result, the proportion of Rubisco to PEPC and PPDK increases concomitantly (Sugiyama et al., 1984; Sugiharto et al., 1990). The reason for the inconsistency may be that, in young leaves at seedling stage, the nitrogen-dependent changes in the amount of Rubisco, PEPC, and PPDK in plants is mainly due to changes in the rates of protein synthesis (Sugiharto et al., 1990). While in the senescing leaves such as the ear-leaf in grain filling phase in the present study, change of these proteins should be mainly due to the breakdown of these proteins (Feller et al., 2008; Masclaux-Daubresse et al., 2010). In fact, in the old leaves of maize seedlings, N starvation reduced Rubisco, PEPC, and PPDK to a similar extent (Sugiharto et al., 1990). The above data suggest that, in N-deficient functional leaves at seedling stage, maize maintains photosynthesis system probably by downregulating CO_2_-trapping mediated by PEPC and PPDK but maintaining CO_2_ conversion mediated by Rubisco. This is supported by Usuda (1984) who found that Rubisco but not PEPC may be rate-limiting factors for photosynthesis in maize seedling. Similarly, Usuda et al. (1984) find that, among ten C_4_ plants, photosynthetic rate was strongly correlated to Rubisco but not PEPC activity. While in the mature leaves in which a large amount of soluble proteins (including Rubisco, PEPC, and PPDK) accumulate (**Table 4**, Sugiharto et al., 1990; Lawlor, 2002), the amount of Rubisco, PEPC, and PPDK might not be the limiting factors for maintaining photosynthesis. Other factors, such as electron transfer and quantum yield as suggested above might become essential for CO_2_ assimilation.

Yin et al. (2011) found that maize plants grown under low N had lower bundle-sheath conductance (g_bs_) for CO_2_, which contributed to their lower CO_2_ leakage and photorespiration. The lower g_bs_ value under low N was presumably associated with an increase in wall thickness of the bundle-sheath cells (Leegood, 2002; Yin et al., 2011). In the present study, the fraction of cell wall biomass was increased by low-N stress, probably because of enhanced carbohydrate accumulation in leaves and allocation to cell walls (Schlüter et al., 2012). Although N_cw_ decreased under LN treatment, the percentage of N_cw_ in total leaf N remained unchanged (**Table 4**; **Figure 3**). This result suggests that N allocation to cell walls is preserved to maintain normal cell wall properties under LN conditions, thus possibly contributing to a decrease in g_bs_ and maintenance of *P*_n_ in bundle sheath cells to the greatest extent possible.

Leaf proteins and cell walls together constituted 72 and 78% of leaf N in LN and HN-grown plants, respectively (**Figure 3**). The remaining N would have included nucleic acids and defensive compounds such as alkaloids, cyanogenic glycosides, and nicotine (Onoda et al., 2004; Takashima et al., 2004). Nucleic acids account for approximately 5–15% of leaf N (Chapin et al., 1986; Chapin, 1989; Evans, 1989b), while defensive compounds constitute up to about 5% (Höft et al., 1996; Burns et al., 2002). N investment into these N components was maintained under low-N conditions (**Figure 3**), suggesting these components are crucial for normal physiological activity under low-N stress.

Yield increase during Green Revolution is largely driven by the increases of the portion of biomass partition into grains. Most recently, it is supposed that redesigning photosynthesis is essential to sustainably meet global food and bioenergy demand, that is, increase photosynthesis with less input of land, water, nutrients etc. (Evans, 2013; Ort et al., 2015). New models are proposed to increase the efficiency of light capture, light energy conversion, carbon capture and conversion, possibly by fast developing genetic engineering technologies (Ort et al., 2015). During grain filling phase in field grown maize, N is continuously remobilized from the leaves and transported to grains for protein synthesis. In such a situation, the current results suggest that sufficient N allocation into bioenergetics is essential to maintain leaf photosynthesis with reducing SLN. That means, under normal N supplies, the amount of Rubisco, PEPC, and PPDK might be beyond what is required for achieving the potential *P*_n_ (Sugiyama et al., 1984; Tazoe et al., 2005; Uribelarrea et al., 2009). Therefore, if grain protein is not a concern, N partitioning into Chl, light-harvesting proteins, and soluble protein (including Rubisco, PEPC, and PPDK) etc., can be substantially reduced to improve N utilization efficiency. On the other hand, the current data also imply that, to further increase the potential of leaf photosynthesis at optimal growth condition, genetic modification of Rubisco, PEPC, and PPDK should focus more on their activity, but not their synthesis. For example, Uribelarrea et al. (2009) found a positive linear correlation between Rubisco initial activity and net photosynthesis in maize at grain filling stage. Ghannoum et al. (2005) concluded that superior N-use efficiency of NADP-ME relative to NAD-ME grasses is related to faster Rubisco turn over. Further studies are required to verify these hypotheses.

## Conclusion

Optimization of N allocation within maize leaves may be an adaptive mechanism to maximize N utilization for photosynthesis, thus increasing grain yield per unit of plant N during the grain-filling stage under a low-N environment. Although ear-leaf SLN was reduced by 38% under LN treatment, *P*_n_ was hardly affected and PNUE was increased by 54%. Under low-N stress, maize plants tended to invest relatively more N into bioenergetics to sustain electron transport. In contrast, Chl and light-harvesting proteins were reduced to control excess electron production. Under low N, total soluble protein per unit leaf area and the proportion of N allocated to soluble protein were reduced by 49 and 19%, respectively, suggesting that a large portion of soluble protein served as N storage reservoirs to be later remobilized to grain for protein synthesis in HN treatment. Among the three major enzymes—Rubisco, PEPC, and PPDK —the percentage of leaf N allocated to Rubisco, PEPC, and PPDK declined 24, 28, and 23%, respectively, under LN treatment. These suggested that these proteins are present in even greater excess for photosynthesis under normal N supply. Our findings suggest that PNUE and whole-plant N utilization efficiency can be increased in maize by optimizing N partitioning within leaves.

## Author Contributions

XM collected the samples, analyzed the samples, and drafted the manuscript. QC made a contribution to acquisition and analysis of the work. FC and LY made a contribution to design of the work. GM made a contribution to design of the work, analysis and revise the manuscript.

## Conflict of Interest Statement

The authors declare that the research was conducted in the absence of any commercial or financial relationships that could be construed as a potential conflict of interest.

## Supplementary Material

FIGURE S1 **Effect of nitrogen (N) supply on the amounts of phosphoenolpyruvate carboxylase (PEPC), pyruvate orthophosphate dikinase (PPDK), and the Rubisco large subunit (LSU) in maize ear-leaves as revealed by sodium dodecyl sulfate-polyacrylamide gels electrophoresis (Tazoe et al., 2005).** Leaves were sampled during the grain-filling stage in maize grown under 0 and 180 kg ha^-1^ supplied N. Four biological replicates are shown per N treatment.
