# Supplementary material for: Within-Leaf Nitrogen Allocation in Adaptation to Low Nitrogen Supply in Maize during Grain-Filling Stage
Source: Front Plant Sci. 2016 May 24;7:699. doi: 10.3389/fpls.2016.00699 (PMC4877366; doi:10.3389/fpls.2016.00699)
Supplement: Supplementary file 1 [file Table_1.DOCX]

Table 1S. Significance level of the fixed effects for each of the measured variables of the ear-leaf of maize during grain filling stage.

| Measured variable | Source of variation | | |
| --- | --- | --- | --- |
|  | N | Year | NⅹYear |
| Cell wall biomass fraction | 0.0001 | ns | ns |
| Chl | 0.0001 | ns | ns |
| Chl a | 0.0001 | ns | ns |
| Chl a/b ratio | 0.0001 | 0.004 | ns |
| Chl b | 0.0001 | 0.042 | ns |
| ETR | ns | ns | ns |
| Grain yield | 0.0001 | ns | ns |
| N accumulation in maturity | 0.0001 | 0.001 | ns |
| N_a_ | 0.0001 | 0.006 | 0.044 |
| N_b_ | ns | 0.0001 | ns |
| N_cw_ | 0.0001 | ns | 0.009 |
| N_h_ | 0.0001 | ns | ns |
| N_others_ | ns | 0.024 | ns |
| Nitrate | 0.003 | 0.002 | 0.035 |
| N_TH_ | 0.0001 | 0.0001 | ns |
| NUtE | 0.0001 | 0.014 | ns |
| Organic N | 0.0001 | 0.022 | ns |
| PEPC | 0.0001 | ns | ns |
| P_n_ | ns | 0.011 | ns |
| PNUE | 0.0001 | 0.001 | ns |
| PPDK | 0.0001 | 0.003 | ns |
| Rubisco | 0.0001 | ns | 0.0001 |
| SLN | 0.0001 | 0.026 | ns |
| Solube protein | 0.0001 | 0.0001 | ns |
| ΦPSII | ns | ns | ns |

ns: not signiﬁcant (*P*> 0.05).
