# Supplementary figures and images for: Within-Leaf Nitrogen Allocation in Adaptation to Low Nitrogen Supply in Maize during Grain-Filling Stage
Source: Front Plant Sci. 2016 May 24;7:699. doi: 10.3389/fpls.2016.00699 (PMC4877366; doi:10.3389/fpls.2016.00699)

1  
2

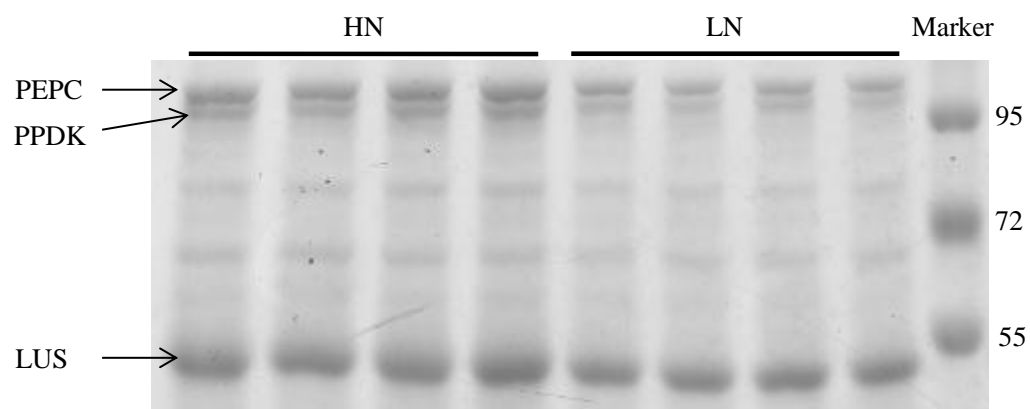

3  
4  
5  
6  
7  
8  
9

Figure 1S

Supplement: FIGURE S1 — Effect of nitrogen (N) supply on the amounts of phosphoenolpyruvate carboxylase (PEPC), pyruvate orthophosphate dikinase (PPDK), and the Rubisco large subunit (LSU) in maize ear-leaves as revealed by sodium dodecyl sulfate-polyacrylamide gels electrophoresis (Tazoe et al., 2005). Leaves were sampled during the grain-filling stage in maize grown under 0 and 180 kg ha-1 supplied N. Four biological replicates are shown per N treatment. [file Image_1.PDF]
